# Prognostic impact of soluble PD-L1 derived from tumor-associated macrophages in non-small-cell lung cancer

**DOI:** 10.1007/s00262-023-03527-y

**Published:** 2023-08-30

**Authors:** Koji Teramoto, Tomoyuki Igarashi, Yoko Kataoka, Mitsuaki Ishida, Jun Hanaoka, Hidetoshi Sumimoto, Yataro Daigo

**Affiliations:** 1 Department of Medical Oncology and Cancer Center, Shiga University of Medical Science, Seta-Tsukinowa, Otsu, Shiga 520-2192 Japan; 2 Center for Advanced Medicine Against Cancer, Shiga University of Medical Science, Seta-Tsukinowa, Otsu, Shiga, 520-2192 Japan; 3 Department of Surgery, Shiga University of Medical Science, Seta-Tsukinowa, Otsu, Shiga 520-2192 Japan; 4 Department of Pathology and Laboratory Medicine, Kansai Medical University, 2-5-1, Shin-machi, Hirakata, Osaka 573-1010 Japan; 5 Institute of Medical Science, The University of Tokyo, 4-6-1, Shirokanedai, Minato-ku, Tokyo 108-8639 Japan; 6 Present Address: Department of Pathology, Osaka Medical and Pharmaceutical University, 2-7, Daigaku-machi, Takatsuki, 569-8686 Osaka Japan

**Keywords:** Soluble PD-L1, Non-small cell lung cancer, Tumor-associated macrophage, Surgery, relapse-free survival

## Abstract

Programmed cell death-ligand 1 (PD-L1) on tumor cells can be degraded to soluble form (sPD-L1) and enter circulation, however, the clinical significances of sPD-L1 in peripheral blood remains to be elucidated in non-small-cell lung cancer (NSCLC). We monitored plasma sPD-L1 levels during perioperative periods and evaluated PD-L1-positive cells in tumor tissues in patients with operable NSCLC. Then the correlation between preoperative plasma sPD-L1 levels and relapse-free survival (RFS) was analyzed retrospectively. In patients who underwent radical surgery (*n* = 61), plasma sPD-L1 levels (median; 63.5 pg/mL) significantly increased 1 month after surgery (72.2 pg/mL, *P* < 0.001). The combined score of PD-L1-positive cells including tumor cells and tumor-associated macrophages (TAMs) was significantly associated with preoperative plasma sPD-L1 levels. In patients with high levels of preoperative plasma sPD-L1, the probability of 5-year RFS was significantly poor for patients with low PD-L1 expression intensity of tumor cells (tcPD-L1) compared with those with high tcPD-L1 (33.3% *vs.* 87.5%, respectively, *P* = 0.016; 95% CI, 0.013–0.964). In former group, PD-L1-positive TAMs were markedly infiltrating compared with those from latter group (246.4 *vs.* 76.6 counts/mm^2^, respectively, *P* = 0.003). In NSCLC, plasma sPD-L1 can reflect the accumulation of PD-L1-posotive TAMs, not just PD-L1-positive tumor cells. In patients with high levels of preoperative plasma sPD-L1, the prognoses after surgery depends on which PD-L1-positive cells, tumor cells or TAMs, are the primary source of the sPD-L1. Thus, measuring both plasma sPD-L1 levels and PD-L1 expression status of tumor cells and TAMs is of benefit for assessment of postoperative prognosis in operable NSCLC.

**Supplementary Information:**

The online version contains supplementary material available at 10.1007/s00262-023-03527-y.

## Introduction

Since the emergence of immune checkpoint inhibitors as one of the standard therapeutics for non-small cell lung cancer (NSCLC) [1–4], there has been increasing interest in the clinical significance of these molecules responsible for immune checkpoint mechanisms in NSCLC. Programmed cell death-ligand 1 (PD-L1) is expressed on cancer cells and interacts with programmed cell death-1 (PD-1) which is expressed on activated lymphocytes, thereby inducing exhaustion and apoptosis of the activated lymphocytes [5, 6]. Thus, through the PD-1/PD-L1 interaction, cancer cells are believed to suppress antitumor immune responses.

In patients with NSCLC, measuring the percentage of PD-L1-positive cancer cells in cancer tissues by immunohistochemistry (IHC) is useful for predicting the efficacy and application of anti-PD-1 and -PD-L1 therapeutic antibodies [7–9]. In this context, the PD-L1 expression status of NSCLC cells has been a focus of several studies. We have also reported on the correlation between the PD-L1 expression status of NSCLC cells and relapse-free survival (RFS) after radical surgery in patients with NSCLC [10]. As we studied PD-L1 expression in NSCLC, we also hypothesized that the PD-L1 molecule in cancer cells would eventually flow into the circulation, given that it is degraded into a soluble form by matrix metalloproteinase-mediated proteolytic cleavage [11, 12] and presumably through cytotoxicity-mediated antitumor immune responses.

Previous studies reported that soluble PD-L1 (sPD-L1) could be detected in the peripheral blood of NSCLC patients [13–20], however, some studies have reported no correlation between sPD-L1 levels in peripheral blood and PD-L1 expression in NSCLC cells [13, 14]. On the basis of those data, we next wondered what cells other than cancer cells could be the source of sPD-L1 in the peripheral blood. If the sPD-L1 in peripheral blood were derived not only from cancer cells, we hypothesized that other cell types should exist that are both a source of sPD-L1 and contribute to poor prognosis in patients with NSCLC. Thus, the group of patients with high sPD-L1 levels in peripheral blood could likely be divided into several subgroups depending on the sources of sPD-L1.

To reveal the biological and clinical significances of sPD-L1 in peripheral blood and answer these questions, in this study, we measured sPD-L1 levels in peripheral blood in settings that were different from previous studies. We compared plasma sPD-L1 levels before and after surgery in patients with NSCLC, meaning a comparison of sPD-L1 levels in the presence and absence of tumors. Additionally, we investigated association of preoperative plasma sPD-L1 with RFS after surgery, and explored the PD-L1-expressing cell type that could influence the prognosis of patients.

## Materials and methods

### Patients and clinical samples

In total, 69 patients who underwent surgery for pulmonary tumors at Shiga University of Medical Science Hospital between November 2013 and October 2015 were enrolled in this study. Among them, 63 patients were pathologically diagnosed with NSCLC (Table 1), while the others were diagnosed with benign tumor by postoperative pathological examination. None of the included patients received neoadjuvant chemotherapy or any other antitumor therapy prior to surgery. Peripheral blood samples were obtained from the patients within 1 week of surgery and 1 and 3 months after surgery; all samples were heparinized to collect mononuclear cells for another study. Plasma samples were isolated by centrifugation and stored at − 80℃ until use. Tumor tissue samples for immunohistochemistry (IHC) were obtained from resected specimens and processed using standard formalin fixation/paraffin embedding protocols. Clinicopathological data were obtained from patient medical records. This study was approved by the Research Ethics Committee of Shiga University of Medical Science (Approval no.: R2013-096) and was performed in accordance with the Declaration of Helsinki. Written informed consent was obtained from all patients who provide samples.

**Table 1:** Patients' characteristics (*n* = 63)

Age (y.o.) (median, range)	70, 49–83
Gender, *n* (%)	
Male	45 (71.4)
Female	18 (28.6)
Smoking habits, *n* (%)	
Never	16 (25.4)
Current/formaer	47 (74.6)
Pathology, *n* (%)	
Adenocarcinoma	43 (68.2)
Squamous cell ca	17 (27.0)
Adeno-squamous cell ca	1 (1.6)
Pleomorphic	2 (3.2)
Tumor size (mm) (median, range)	25, 9–137
Cell grade, *n* (%)	
Grade 1	28 (44.4)
Grade 2	19 (30.2)
Grade 3	16 (25.4)
Pathological stage, *n* (%)	
1A	36 (57.1)
1B	11 (17.5)
2A	5 (7.9)
2B	0 (0.0)
3A	9 (14.3)
4	2 (3.2)
Invasion to lymphatics, *n* (%)	27 (42.9)
Invasion to microvessels, *n* (%)	42 (66.7)

### PD-L1 ELISA

The levels of sPD-L1 in plasma and culture supernatant samples were measured by ELISA using Human PD-L1 ELISA Kit (clone of anti-PD-L1 antibody: 28–8, Abcam, Cambridge, UK). The 75th percentile value in the investigated cohort was used as the cutoff value, and a high level of plasma sPD-L1 was defined as cases where the level was higher than the cutoff.

### PD-L1 immunohistochemistry

Whole tumor Sects. (4-μm-thick) of formalin-fixed paraffin-embedded tissue were deparaffinized in xylene and rehydrated in ethanol and distilled water. Antigen retrieval was performed by microwaving sections in Universal HIER antigen retrieval reagent (Abcam) for 20 min. Endogenous peroxidase activity was blocked by treatment with Peroxidase Block (DAKO, Santa Clara, CA, USA) for 10 min, and non-specific binding was blocked by treatment with Protein Block serum-free Ready-to-use (DAKO) for 10 min at room temperature. The sections were then incubated overnight at 4 °C with anti-human PD-L1 monoclonal antibody (clone 28–8, 1:500; Abcam). The sections were then incubated with Rabbit-specific IHC polymer detection kit and visualized using DAB substrate (both Abcam). Finally, sections were counterstained with hematoxylin. For negative control staining, the anti-PD-L1 primary antibody was replaced with a rabbit IgG monoclonal antibody (Abcam).

### Evaluation of PD-L1-expressing cells

To evaluate the PD-L1 expression status of NSCLC cells following IHC staining, we used a semi-quantitative scoring method that reflects both the intensity and extent of PD-L1 expression of tumor cells and is expressed as the PD-L1 expression score (H-score), as described previously [21]. Briefly, PD-L1 staining on tumor cells was scored relative to that on alveolar macrophages in the same section, with score 0, 1, 2, and 3 corresponded to no staining, weak staining (tumor cell intensity lower than alveolar macrophages), moderate staining (tumor cell intensity similar to that of alveolar macrophages), and strong staining (tumor cell intensity stronger than that of alveolar macrophages), respectively. The total number of tumor cells was counted in three randomly selected fields under 200 × magnification, and the percentage of PD-L1-stained tumor cells was calculated. The final H-score was calculated as:

$$\left( {\left[ {{1 } \times \, \% {\text{ of cells scoring 1}}} \right] \, + \, \left[ {{2 } \times \, \% {\text{ of cells scoring 2}}} \right] \, + \, \left[ {{3 } \times \, \% {\text{ of cells scoring 3}}} \right]} \right)$$

To evaluate the cell density of PD-L1-positive TAMs, the total number of PD-L1-positive TAMs was counted in three randomly selected fields under 400 × magnification, and the cell density of PD-L1-positive TAMs was calculated for each field of view by dividing the cell counts by the area of the field of view. Sections were independently examined by two researchers, including a pathologist, and the average of the cell densities in each field of view was calculated.

### Generation of macrophages in vitro

Human CD14-positive peripheral blood mononuclear cells (PBMCs) (Lonza, Köln, Germany) were cultured in AIM-V medium (Life Technologies, Grand Island, NY, USA) in the presence of granulocyte macrophage colony-stimulating factor for Type 1 (M1) and macrophage colony-stimulating factor for Type 2 (M2) macrophages (CellVivo Human M1 and M2 Macrophage Differentiation Kit, R&D, Minneapolis, MN, USA) for 6 d. Then, the cells were stimulated with 1 μg/mL lipopolysaccharide for 24 h to generate activated M1 and M2 cells. The culture supernatants were collected, and levels of sPD-L1 were measured by ELISA as described above. The expression levels of specific markers for each type of macrophage were evaluated by flow-cytometry using a BD FACS Calibur, and the data were analyzed using BD CellQuest Pro software (BD Biosciences, San Jose, CA, USA). Antibodies used for detection were PE mouse anti-CD80 (clone: L307.4, BD Biosciences) and anti-CD229 (interferon-gamma receptor) (clone: GIR-208, Life Technologies, Carlsbad, CA, USA) for M1, PE mouse anti-CD163 (clone: 215934, R&D) and anti-CD206 (clone: 19.2, BD Biosciences) for M2, and PE mouse anti-PD-L1 antibody (clone: MIH1, BD Biosciences) for PD-L1.

### Statistical analysis

For continuous variables, comparisons between two groups were analyzed using the Mann–Whitney *U* test, and comparisons between more than three groups were analyzed using ANOVA. Pearson’s product-moment correlation coefficient was used to analyze the correlation between two continuous variables. Relapse-free survival (RFS) after surgery was calculated using Kaplan–Meier analysis and was compared with the log-rank test. All analyses were performed using SPSS Statistics 25.0 software (IBM, Armonk, NY, USA).

## Results

### Correlations of preoperative plasma sPD-L1 levels with PD-L1 expression on tumor cells

**Fig. 1:**
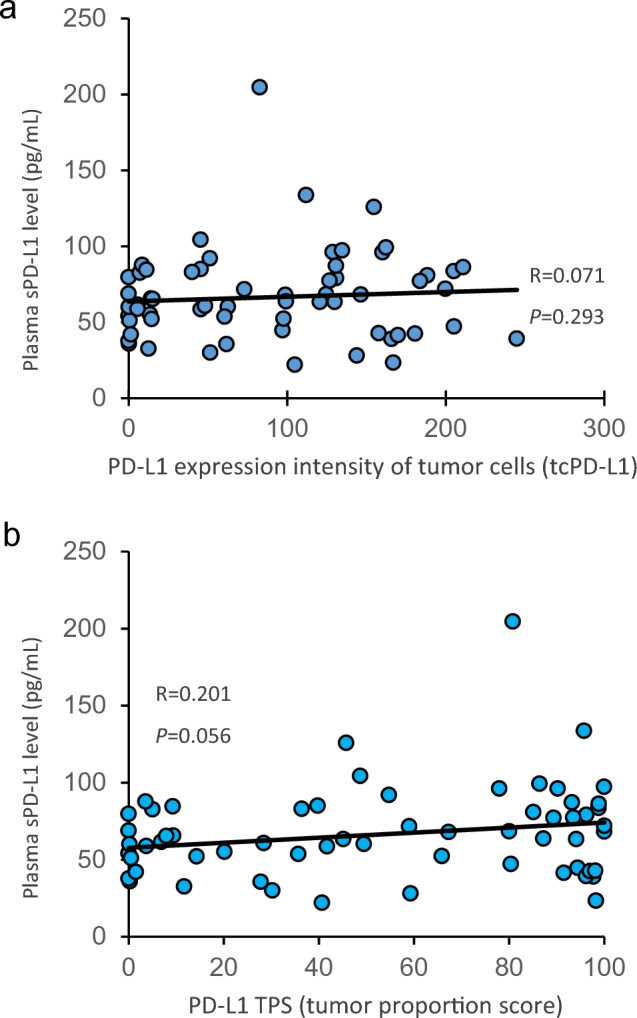
In patients with non-small cell lung cancer (*n* = 61), correlations of preoperative plasma soluble PD-L1 levels measured by ELISA with **a** PD-L1 expression intensity on tumor cells and **b** PD-L1 tumor proportion score determined followed by immunohistochemistry

Preoperative plasma sPD-L1 levels were measured for 63 patients with NSCLC (Supplementary Table 1), and the median plasma sPD-L1 level was 63.6 pg/mL, with a range of 0.0 to 204.6 pg/mL. Using the H-score as a semi-quantitative evaluation of the expression intensity of PD-L1 on tumor cells (tcPD-L1), the median tcPD-L1 expression score was 97.1, with a range of 0.0 to 244.9. Then, we analyzed the correlation between preoperative plasma sPD-L1 levels and tcPD-L1 expression score. However, a significant correlation was not observed between two (*R* = 0.0071, *P* = 0.293) (Fig. 1a). In addition, we measured the proportion of PD-L1-positive tumor cells (PD-L1 TPS), yielding the median PD-L1 TPS of 52.1% with a range of 0.0 to 100%. Then, we analyzed the correlation between preoperative plasma sPD-L1 levels and PD-L1 TPS, and the data demonstrated that However, preoperative plasma sPD-L1 levels tended to correlate with PD-L1 TPS (*R* = 0.201, *P* = 0.056) (Fig. 1b). However, on the whole, the data suggest that sPD-L1 in peripheral blood is not necessarily derived from tcPD-L1.

### Correlations of preoperative plasma sPD-L1 levels with clinicopathological factors

Next, we analyzed correlations between preoperative plasma sPD-L1 levels and clinicopathological factors (Supplementary Fig. 1). The data demonstrated that preoperative plasma sPD-L1 levels were significantly higher in male compared with female patients (median: 65.7 *vs.* 42.6 pg/mL, *P* = 0.025) and in smokers compared with never smokers (68.3 *vs.* 47.2 pg/mL, *P* = 0.032). However, sPD-L1 levels were not associated with age (R = 0.121, *P* = 0.174), tumor size (R = -0.012, *P* = 0.467), pathological type (median: 65.5, 58.9, 55.2 and 82.1 pg/mL in adeno, squamous cell, adeno-squamous cell and pleomorphic carcinoma, respectively, *P* = 0.711), postoperative pathological stage (median: 73.8, 68.1, 58.9, 61.3 and 91.2 pg/mL in IA, IB, IIA, IIIA and IV, respectively, *P* = 0.669), tumor cell grade (median, 60.3, 717 and 65.7 pg/mL in Grade 1, 2, and 3, respectively, *P* = 0.121), lymphatic invasion (median: 62.5 *vs.* 64.7 pg/mL, *P* = 0.549), or microvessel invasion of tumor cells (median: 65.4 *vs.* 63.4 pg/mL, *P* = 0.716).

### Perioperative changes in sPD-L1 levels in peripheral blood

**Fig. 2:**
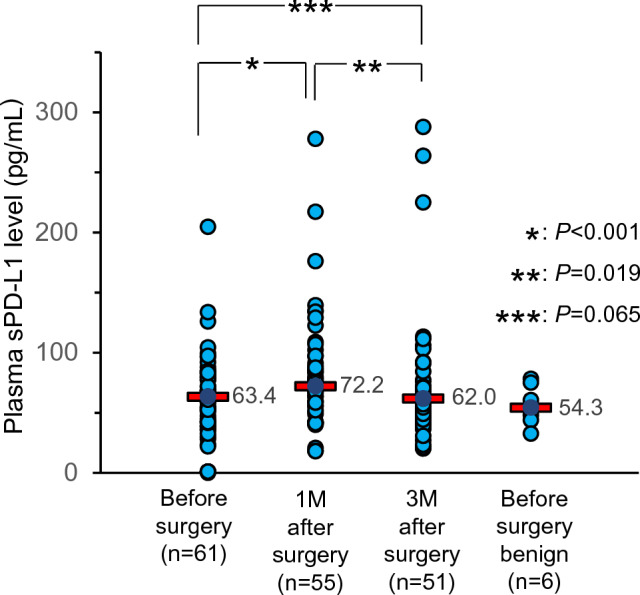
Changes in plasma soluble PD-L1 (sPD-L1) levels in plasma during the perioperative period in patients who underwent radical surgery, including lobectomy and dissection of regional lymph nodes, for non-small cell lung cancer. Plasma sPD-L1 levels were measured by ELISA before surgery (*n* = 56), 1 month (*n* = 53), and 3 months (*n* = 50) after surgery. The data from patients with benign pulmonary tumor are plotted (*n* = 6). Red bars indicate the median sPD-L1 levels in plasma

Preoperative plasma sPD-L1 levels were suggested not to be associated with tcPD-L1 expression. In this context, to rule out the possibility that only PD-L1-expressing tumor cells contributed to sPD-L1 levels in peripheral blood, we examined postoperative plasma sPD-L1 levels in patients who underwent radical surgery including lobectomy or segmentectomy and lymph node dissection (*n* = 61) (Supplementary Table 1). The median preoperative serum sPD-L1 level was 63.4 pg/mL, and 1 month after surgery, the median sPD-L1 level (*n* = 55) had significantly increased to 72.2 pg/mL (*P* < 0.001) (Fig. 2). Furthermore, at 3 months after surgery, the median serum sPD-L1 level (*n* = 51) had significantly decreased to the preoperative level (62.0 pg/mL, *P* = 0.019) (Fig. 2). These data demonstrated that despite completely removing tumor cells, plasma sPD-L1 levels were temporarily increased and then subsequently recovered to the initial levels, suggesting that some factor(s) other than tumor cells, such as an inflammatory immune response, might contribute to sPD-L1 levels in peripheral blood.

### PD-L1-positive tumor-associated macrophages

**Fig. 3:**
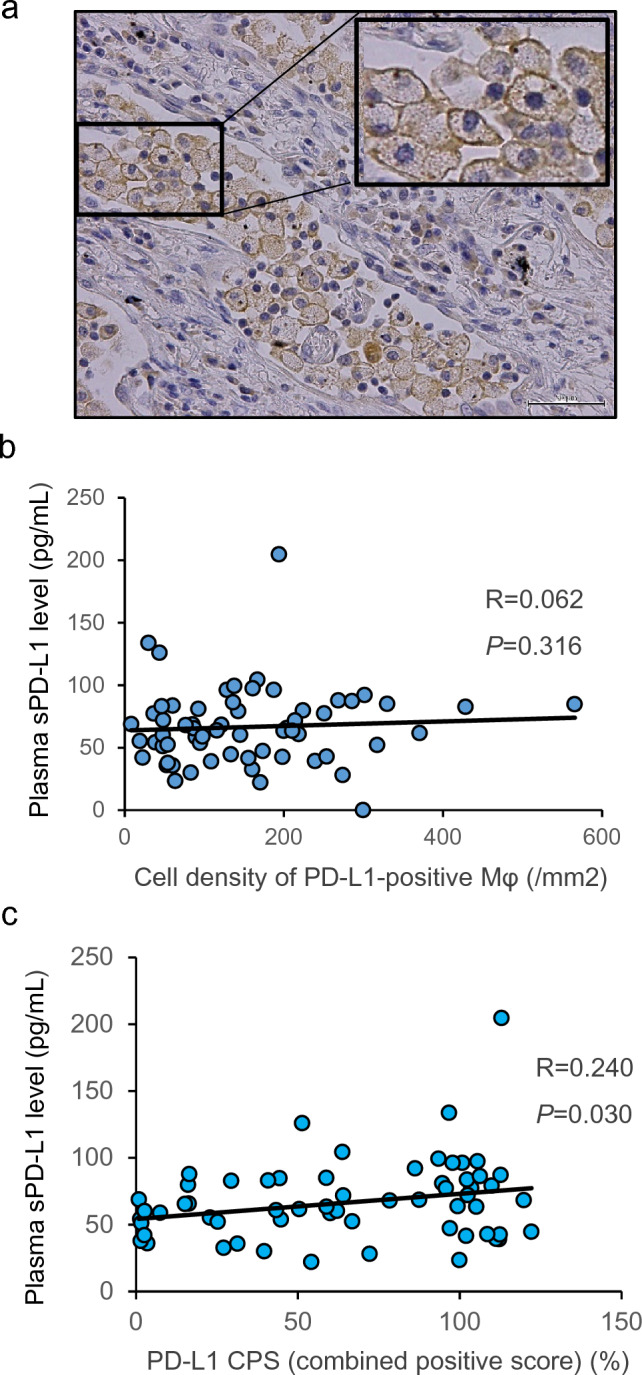
**a** Through immunohistochemistry, tumor-associated macrophages (TAMs) were positive for PD-L1 and infiltrated to tumor tissue; original magnification: 400 × , scale bar: 50 μm. In patients with non-small cell lung cancer (*n* = 61), correlations of preoperative plasma soluble PD-L1 levels measured by ELISA with **b** cell density of PD-L1-positive macrophages in tumor tissues and (**c)** PD-L1 combined positive score focusing on PD-L1-positive tumor cells and macrophages determined though immunohistochemistry

Our searching for PD-L1-positive cells in NSCLC tumor tissues included immunohistochemistry, through which we found that tumor-associated macrophages (TAMs) were positive for PD-L1, especially those that had gathered in specific regions (Fig. 3a). On the basis of these findings, we focused on PD-L1-positive TAMs and measured the cell density of these cells. Then, we analyzed the correlation between preoperative plasma sPD-L1 levels and the cell density of PD-L1-positive TAMs. As results, the data demonstrated that no significant correlation was observed between two (*R* = 0.062, *P* = 0.316) (Fig. 3b). However, when we measured the combined positive score focusing on PD-L1-positive tumor cells and PD-L1-positive TAMs (PD-L1 CPS), yielding the median PD-L1 CPS of 64.0% with a range of 0.9 to 122.2%. Then, we analyzed the correlation between preoperative plasma sPD-L1 levels and PD-L1 CPS, and the data demonstrated that preoperative plasma sPD-L1 levels significantly correlated with PD-L1 CPS (*R* = 0.240, *P* = 0.030) (Fig. 3c). These data indicate that sPD-L1 levels in peripheral blood can be derived from PD-L1-positive tumor cells as well as PD-L1-positive TAMs.

### Preoperative plasma sPD-L1 levels and RFS

**Fig. 4:**
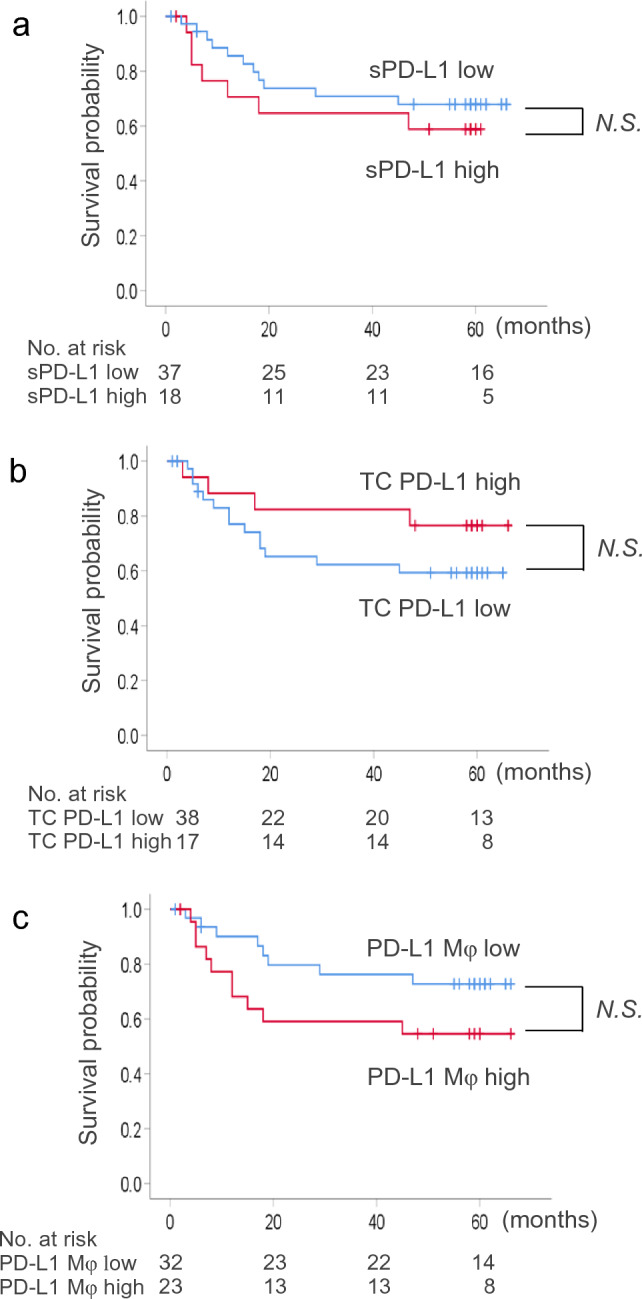
In patients who underwent radical surgery, including lobectomy and dissection of regional lymph nodes, for invasive non-small cell lung cancer with pathological stages of IA to IIIA (*n* = 55), **a** correlation between preoperative plasma soluble PD-L1 (sPD-L1) levels and relapse-free survival (RFS) after surgery analyzed by the Kaplan–Meier method. Red and blue lines indicate data from patients with high (≥ 80 pg/mL) and low (< 80 pg/mL) plasma preoperative sPD-L1 levels, respectively. **b** Correlation between the PD-L1 expression intensity of tumor cells and RFS after surgery analyzed by the Kaplan–Meier method. Red and blue lines in the indicate data from patients with high (H-score ≥ 150) and low (H-score < 150) PD-L1 expression intensity of tumor cells, respectively. **c** Correlation between the cell density of PD-L1-positive tumor-associated macrophages (TAMs) and RFS after surgery analyzed by the Kaplan–Meier method. Red and blue lines in the indicate data from patients with high (≥ 150/mm^2^) and low (< 150/mm^2^) cell density of PD-L1-positive TAMs, respectively

We investigated the clinical significance of plasma sPD-L1 levels focusing on the origin of sPD-L1 in peripheral blood. First, patients who underwent radical surgery for invasive carcinoma (*n* = 55) (Supplementary Table 2) were classified into two groups according to preoperative plasma sPD-L1 levels: the high sPD-L1 group (≥ 80 pg/mL) and the low sPD-L1 group (< 80 pg/mL), as described earlier. Kaplan–Meier analysis revealed that postoperative RFS tended to be shorter in the high sPD-L1 group (*n* = 18) than in the low sPD-L1 group (*n* = 37), which had 5-year relapse-free probabilities of 58.8% and 67.9%, respectively (*P* = 0.442, 95% confidence interval [CI]: 0.268–1.785) (Fig. 4a).

Next, we analyzed the correlation between tcPD-L1 expression intensity and RFS after surgery. Patients were classified into two groups according to the tcPD-L1 expression intensity: the high tcPD-L1 expression group (≥ 150) and the low tcPD-L1 expression group (< 150), as in a previous report [10]. Kaplan–Meier analysis revealed that postoperative RFS tended to be longer for the high tcPD-L1 expression group (*n* = 17) than for the low tcPD-L1 expression group (*n* = 38), which had 5-year relapse-free probabilities of 76.5% and 59.3%, respectively (*P* = 0.241, 95% CI: 0.171–1.587) (Fig. 4b). In contrast with the data of plasma sPD-L1, high tcPD-L1 expression intensity tended to be better prognosis after surgery in NSCLC, which is compatible with our previous data [10].

In addition, we analyzed the correlation between the cell density of PD-L1-positive TAMs and RFS after surgery. Patients were classified into two groups according to the cell density of PD-L1-positive TAMs: the high PD-L1-positive TAMs group (≥ 150/mm^2^) and the low PD-L1-positive TAMs group (< 150/ mm^2^) (Area under the curve = 1.000). Kaplan–Meier analysis revealed that postoperative RFS tended to be longer for the low PD-L1-positive TAMs group (*n* = 30) than for the high PD-L1-positive TAMs group (*n* = 25), which had 5-year relapse-free probabilities of 72.8% and 54.5%, respectively (*P* = 0.241, 95% CI: 0.196–1.262) (Fig. 4c).

### Different prognoses by the origin of sPD-L1 in peripheral blood

**Fig. 5:**
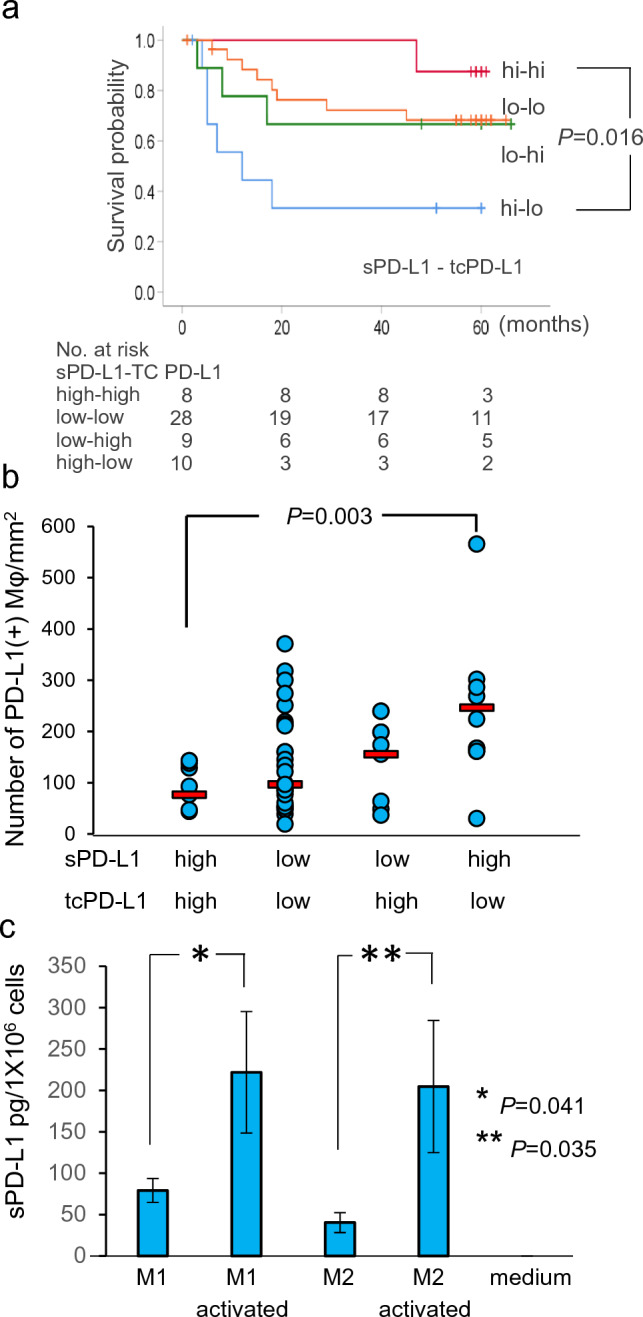
Patients who underwent radical surgery, including lobectomy and dissection of regional lymph nodes, were classified into four groups according to preoperative plasma soluble PD-L1 (sPD-L1) levels and the PD-L1 expression intensity of tumor cells, and then **a** relapse-free survival after surgery was analyzed by Kaplan–Meier method. Red and blue lines indicate the data of patients with high preoperative sPD-L1 levels in plasma (≥ 80 pg/mL) and high (H-score ≥ 150) or low (H-score < 150), respectively. Green and orange lines indicate the data from patients with low preoperative sPD-L1 levels in plasma (< 80 pg/mL) and high or low PD-L1 expression intensity of tumor cells, respectively. **b** The cell density of PD-L1-positive tumor-associated macrophages (TAMs). Red bars indicate the median cell density of PD-L1-positive TAMs. **c** The levels of sPD-L1 that were secreted from type 1 and type 2 macrophages. The cells were generated in vitro and activated by lipopolysaccharide, then the sPD-L1 levels in the supernatant were measured by ELISA

Given that the contributions to RFS after surgery may be distinct between sPD-L1 and tcPD-L1, we next classified the patients into four groups according to preoperative plasma sPD-L1 levels and tcPD-L1 expression intensity, and then analyzed RFS after surgery. Kaplan–Meier analysis revealed that postoperative RFS was significantly shorter for patients in the high sPD-L1–low tcPD-L1 group (*n* = 10) compared with those in the high sPD-L1–high tcPD-L1 group (*n* = 8), which had 5-year relapse-free probabilities of 33.3% and 87.5%, respectively (*P* = 0.016, Hazard ratio; 0.114, 95% CI; 0.130–0.964) (Fig. 5a). These data suggest that among NSCLC patients with high preoperative plasma sPD-L1 levels, those whose plasma sPD-L1 was presumably derived from high tcPD-L1 expression might have a better prognosis after surgery, given that postoperative RFS tended to be longer for the high tcPD-L1 expression group than for the low tcPD-L1 expression group (Fig. 4b). In contrast, for NSCLC patients with high preoperative plasma sPD-L1 and low tcPD-L1 expression, PD-L1-positive TAMs must be responsible for the elevated sPD-L1 level in peripheral blood and also contribute to poor prognosis, given that postoperative RFS tended to be shorter for the high PD-L1-positive TAMs group than for the low PD-L1-positive TAMs group (Fig. 4c). In fact, in the high sPD-L1–low tcPD-L1 group (*n* = 10), the cell density of PD-L1-positive TAMs was significantly high compared with that in the high sPD-L1–high tcPD-L1 group (*n* = 8) (median ± SD: 246.4 ± 149.5 *vs.*76.6 ± 48.1 counts/mm^2^, respectively, *P* = 0.003) (Fig. 5b).

Finally, we confirmed that sPD-L1 was released from PD-L1-positive macrophages in vitro. Type 1 (M1) and 2 (M2) macrophages were induced from human PBMCs, and these cells were confirmed to express PD-L1 (Supplementary Fig. 2). ELISA demonstrated that both M1 and M2 cells released sPD-L1 into the culture supernatant, and the levels significantly increased following activation with lipopolysaccharide (*P* = 0.041 and *P* = 0.035, respectively) (Fig. 5c). These data suggest that a high density of PD-L1-positive TAMs could contribute to high plasma sPD-L1 levels and poor prognosis after surgery due to its suppressive activity on antitumor immune responses.

## Discussion

This is the first report to monitor the sPD-L1 levels in peripheral blood during perioperative periods in patients with operable NSCLC, and the data demonstrated that peripheral sPD-L1 levels were elevated 1 month after surgery despite the absence of tumors. Focusing on some PD-L1-positive cell types involved in inflammation, the sPD-L1 levels in peripheral blood were revealed to be derived from PD-L1-positive macrophages, not just PD-L1-positive tumor cells. Patients with high levels of preoperative plasma sPD-L1 were divided into two groups by the origin of sPD-L1. In cases in which the plasma sPD-L1 levels were mainly derived from PD-L1-positive tumor cells, the good postoperative prognoses were expected. On the other hand, in cases in which the plasma sPD-L1 levels were mainly derived from PD-L1-positive TAMs, the postoperative prognoses were poor. Therefore, measuring both plasma sPD-L1 levels and tcPD-L1 expression intensity is of benefit for assessment of postoperative prognosis in patients with operable NSCLC.

Initially, we hypothesized that sPD-L1 levels in peripheral blood would decrease after surgery. However, intriguingly, 1 month after surgery, plasma sPD-L1 levels were significantly elevated. Given that the levels were elevated despite the absence of tumors, we focused on some PD-L1-positive cell types involved in inflammation. We have previously shown that cancer-associated fibroblasts (CAFs), which are a dominant cell type in the stroma, can express PD-L1 following stimulation by interferon-gamma (IFN-γ) and that PD-L1-positive CAFs are present in NSCLC tissues [22]. In that study, we obtained preliminary data that some TAMs are positive for PD-L1. Although a previous study reported that sPD-L1 was not released from macrophages [23]; however, our data revealed that activated M1 and M2 macrophages secrete sPD-L1 into culture supernatant. On the basis that sPD-L1 can be derived from activated macrophages, not limited to TAMs, the increase in plasma sPD-L1 levels at 1 month after surgery could be due to local inflammation in the lung that is associated with surgical invasion. There is no information on the half-life of sPD-L1 in peripheral blood, however, the fact that sPD-L1 levels in peripheral blood decreased at 3 months after surgery may be due to the improvement of local inflammation in the lung.

In addition to the data that plasma sPD-L1 levels were significantly elevated 1 month after surgery, we found no correlation between preoperative plasma sPD-L1 levels and tcPD-L1expression intensity, which is consistent with the results of previous reports [14]. In this regard, it is also unlikely that sPD-L1 levels in peripheral blood reflects only tcPD-L1expression status. Then, we considered the possibility that PD-L1-positive TAMs could be a source of sPD-L1 in peripheral blood. As results, the preoperative plasma sPD-L1 levels were not associated with tcPD-L1expression intensity or the cell density of PD-L1-positive TAMs, however, CPS focusing on PD-L1-positive tumor cells and TMAs was significantly associated with the preoperative plasma sPD-L1 levels. These data suggest that sPD-L1 levels in peripheral blood can be derived from PD-L1-positive tumor cells as well as PD-L1-positive TAMs, reflecting the PD-L1 expression status of tumor cells and tumor stromal cells.

Next, we investigated the clinical significance of sPD-L1 in peripheral blood. Previous papers have reported that high sPD-L1 levels in peripheral blood are associated with poor prognosis in patients with advanced NSCLC (reviewed in ref. 24). In addition, in our study, patients with high preoperative plasma sPD-L1 levels tended to have shorter RFS after surgery. However, when the high sPD-L1 group was further classified into two subgroups by tcPD-L1 expression intensity, it was found that prognosis was significantly different between the subgroups with high and low tcPD-L1 expression. These data demonstrated that RFS after surgery was significantly shorter in the group with high plasma sPD-L1 and low tcPD-L1 expression compared with the group with high plasma sPD-L1 and high tcPD-L1 expression. This may depend on which cells, tumor cells or TAMs, are the primary source of the sPD-L1 in peripheral blood.

We have previously reported that high tcPD-L1 expression is a good prognostic biomarker for patients with early-stage NSCLC [10]. This is because cancer cells need the cytokine IFN-γ to express PD-L1, and IFN-γ is secreted from activated lymphocytes in the tumor microenvironment, suggesting the presence of an activated antitumor immune response behind the high tcPD-L1 expression. In Fig. 5A, 87.5% of patients with a high-level plasma sPD-L1 and high tcPD-L1 expression were shown to be early-stage, stage I, NSCLC. Therefore, in operable patients with a high level of plasma sPD-L1 as a consequence of high tcPD-L1expression, the postoperative prognosis may be better due to the background of an activated antitumor immune response. Conversely, in patients with high plasma sPD-L1 levels and low tcPD-L1 expression, the plasma sPD-L1 can be primarily derived from PD-L1-positive TAMs. A large accumulation of TAMs has been reported to be a poor prognosis in patients with resectable NSCLC [25], which was almost compatible with the data in this study (5-year relapse-free probabilities of 72.8% and 54.5%, respectively) (Fig. 4C). In addition, PD-L1-positive M2-like macrophages exerted an immunosuppressive effect by inhibiting the proliferation and activation of CD8-positive T cells in a PD-L1-dependent fashion [26]. Therefore, in those patients, RFS after surgery ended in being shorter. Given that sPD-L1 in peripheral blood is attributed to PD-L1-positive tumor cells and TAMs, It should be noted that in operable NSCLC patients with high plasma sPD-L1 levels, postoperative prognosis differs depending on its source of sPD-L1, tumor cells or TAMs.

We suggested that PD-L1-positive TAMs contributed to the elevated plasma sPD-L1 levels and poor prognosis. However, there are several limitations to this study. First, the number of PD-L1-positive TAMs in tumor tissues was assessed by their density in the tissue. The sPD-L1 level in peripheral blood can be affected by the product of the cell density of PD-L1-positive TAMs and tumor volume; we did not consider tumor volume. Although we examined correlations between plasma sPD-L1 levels and the density of PD-L1-positive TAMs in combination with maximum tumor diameter, no clear data were obtained (data not shown). Second, as mentioned earlier, there are other PD-L1-positive cells in the tumor stroma [22, 23], and it is unclear to what extent these cells could influence sPD-L1 levels in peripheral blood. Third, the level to which sPD-L1 affects the antitumor immune response is unknown. Given that plasma sPD-L1 levels decreased within 3 months after surgery, without specific stimulation, the effect of sPD-L1 would cease 3 months after surgery. Some studies have reported that sPD-L1 possesses immunosuppressive activity [27, 28]; however, the detailed biological activity of sPD-L1 remains unclear [29].

In conclusion, sPD-L1 levels in peripheral blood were significantly elevated 1 month after surgery compared with preoperative levels despite the absence of tumors. Among patients with high levels of preoperative plasma sPD-L1, those with low tcPD-L1 expression had significantly worse postoperative prognoses than those with high tcPD-L1 expression. In the former group, the cell density of PD-L1-positive TAMs was significantly higher, and suppression of antitumor immune responses by TAMs may be the cause of poor prognosis. The sPD-L1 in peripheral blood can be derived from PD-L1-positive TAMs, not just PD-L1-positive tumor cells. Thus, in patients with operable NSCLC, measuring both plasma sPD-L1 levels and PD-L1 expression status of tumor cells and TMAs is of benefit for assessment of postoperative prognosis.

### Supplementary Information

Below is the link to the electronic supplementary material.

Supplementary file1 (PPTX 96 kb)

Supplementary file2 (PPTX 71 kb)

Supplementary file3 (XLSX 10 kb)

Supplementary file4 (XLSX 10 kb)

## Data Availability

The datasets generated during and/or analyzed during the current study are available from the corresponding author on reasonable request.

## Abbreviations

CAFs: Cancer-associated fibroblasts
CPS: Combined positive score
IHC: Immunohistochemistry
NSCLC: Non-small cell lung cancer
PD-L1: Programmed cell death-ligand 1
PD-1: Programmed cell death 1
sPD-L1: Soluble form of programmed cell death-ligand 1
TAMs: Tumor associated macrophages
tcPD-L1: Programmed cell death-ligand 1 on tumor cells
TPS: Tumor proportion score
RFS: Recurrence-free survival
