# Supplementary material for: Prognostic impact of soluble PD-L1 derived from tumor-associated macrophages in non-small-cell lung cancer
Source: Cancer Immunol Immunother. 2023 Aug 30;72(11):3755–64. doi: 10.1007/s00262-023-03527-y (PMC10576714; doi:10.1007/s00262-023-03527-y)
Supplement: Supplementary file 1 — Supplementary file1 (PPTX 96 kb) [file 262_2023_3527_MOESM1_ESM.pptx]

## Slide 1
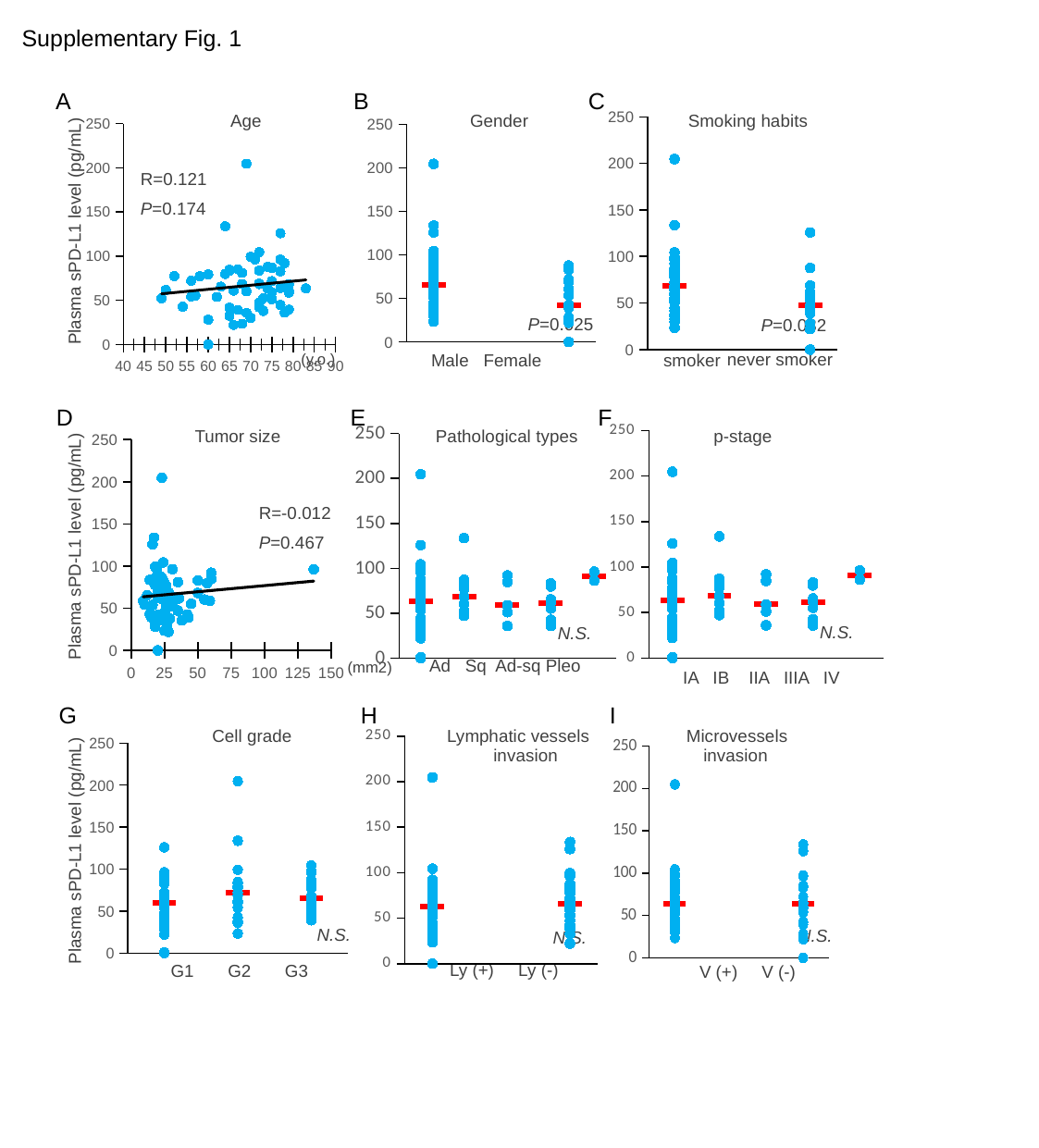

Supplementary Fig. 1
A B C
 Age Gender Smoking habits
### Chart
| Category | | | | | | | | | | | | | | | | | | | | | | | | | | | | | | | | | | | | | | | | | | | | | | | |
|---|---|---|---|---|---|---|---|---|---|---|---|---|---|---|---|---|---|---|---|---|---|---|---|---|---|---|---|---|---|---|---|---|---|---|---|---|---|---|---|---|---|---|---|---|---|---|---|
### Chart
| Category | | | | | | | | | | | | | | | | | | | | | | | | | | | | | | | | | | | | | | | | | | | | | | | |
|---|---|---|---|---|---|---|---|---|---|---|---|---|---|---|---|---|---|---|---|---|---|---|---|---|---|---|---|---|---|---|---|---|---|---|---|---|---|---|---|---|---|---|---|---|---|---|---|
### Chart
| Category | Pre ope |
|---|---|R=0.121
P=0.174
Plasma sPD-L1 level (pg/mL)
P=0.025
P=0.032
 (y.o.)
 Male Female
 never smoker
 smoker
D E F
 Tumor size Pathological types p-stage
### Chart
| Category | | | | | | | | | | | | | | | | | | | | | | | | | | | | | | | | | | | | | |
|---|---|---|---|---|---|---|---|---|---|---|---|---|---|---|---|---|---|---|---|---|---|---|---|---|---|---|---|---|---|---|---|---|---|---|---|---|---|
### Chart
| Category | Pre ope |
|---|---|
### Chart
| Category | | | | | | | | | | | | | | | | | | | | | | | | | | | | | | | | | | | | | |
|---|---|---|---|---|---|---|---|---|---|---|---|---|---|---|---|---|---|---|---|---|---|---|---|---|---|---|---|---|---|---|---|---|---|---|---|---|---|R=-0.012
P=0.467
Plasma sPD-L1 level (pg/mL)
N.S.
N.S.
Ad Sq Ad-sq Pleo
(mm2)
IA IB IIA IIIA IV
G H I
 Cell grade Lymphatic vessels Microvessels
### Chart
| Category | | | | | | | | | | | | | | | | | | | | | | | | | | | | | | | | | | | | | |
|---|---|---|---|---|---|---|---|---|---|---|---|---|---|---|---|---|---|---|---|---|---|---|---|---|---|---|---|---|---|---|---|---|---|---|---|---|---| invasion invasion
### Chart
| Category | | | | | | | | | | | | | | | | | | | | | | | | | | | | | | | | | | | | | | | | | | | | |
|---|---|---|---|---|---|---|---|---|---|---|---|---|---|---|---|---|---|---|---|---|---|---|---|---|---|---|---|---|---|---|---|---|---|---|---|---|---|---|---|---|---|---|---|---|
### Chart
| Category | | | | | | | | | | | | | | | | | | | | | | | | | | | | | | | | | | | | | | | | | | |
|---|---|---|---|---|---|---|---|---|---|---|---|---|---|---|---|---|---|---|---|---|---|---|---|---|---|---|---|---|---|---|---|---|---|---|---|---|---|---|---|---|---|---|Plasma sPD-L1 level (pg/mL)
N.S.
N.S.
N.S.
 Ly (+) Ly (-)
 G1 G2 G3
 V (+) V (-)
