# Supplementary figures and images for: Prognostic impact of soluble PD-L1 derived from tumor-associated macrophages in non-small-cell lung cancer
Source: Cancer Immunol Immunother. 2023 Aug 30;72(11):3755–64. doi: 10.1007/s00262-023-03527-y (PMC10576714; doi:10.1007/s00262-023-03527-y)

## Slide 1
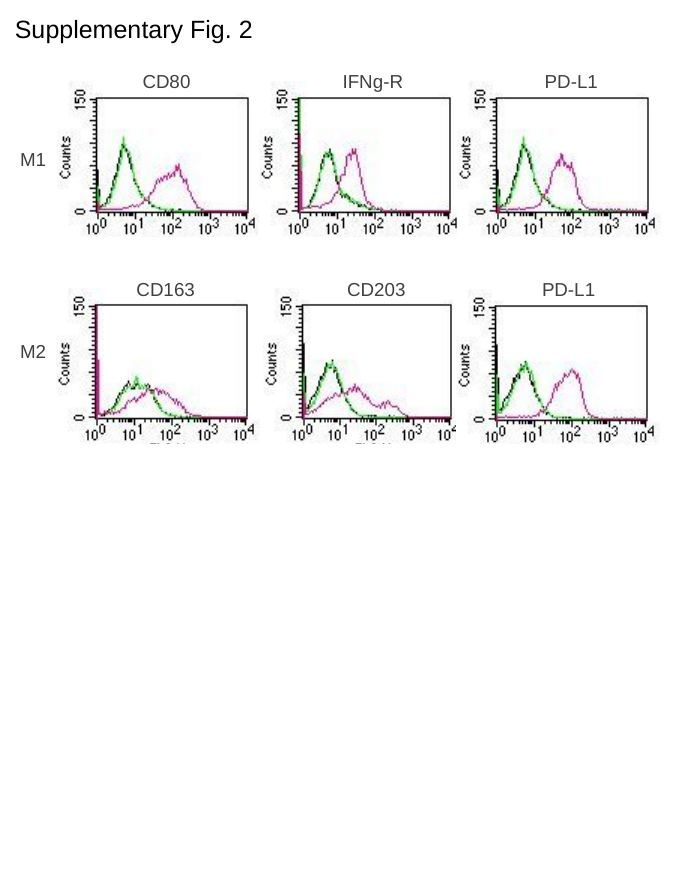

Supplementary Fig. 2
CD80 IFNg-R PD-L1
M1
M2
CD163 CD203 PD-L1

Supplement: Supplementary file 2 — Supplementary file2 (PPTX 71 kb) [file 262_2023_3527_MOESM2_ESM.pptx]
